# Knowledge, attitudes and practices relating to influenza A(H7N9) risk among live poultry traders in Guangzhou City, China

**DOI:** 10.1186/s12879-014-0554-8

**Published:** 2014-10-18

**Authors:** Xiaowei Ma, Qiuyan Liao, Jun Yuan, Yufei Liu, Yanhui Liu, Jiandong Chen, Jianping Liu, Wenfeng Cai, Benjamin J Cowling, Biao Di, Richard Fielding, Ming Wang, Zhicong Yang, Gabriel M Leung, Eric HY Lau

**Affiliations:** Guangzhou Center for Disease Control and Prevention, Guangzhou, 510440 Guangdong Province China; The University of Hong Kong, Hong Kong, China

**Keywords:** Live poultry trader, Avian influenza, Attitudes, Knowledge

## Abstract

**Background:**

Live poultry traders (LPTs) have greater risk to avian influenza due to occupational exposure to poultry. This study investigated knowledge, attitudes and practices of LPTs relating to influenza A (H7N9).

**Methods:**

Using multi-stage cluster sampling, 306 LPTs were interviewed in Guangzhou by a standardized questionnaire between mid-May to June, 2013. Hierarchical logistic regression models were used to identify factors associated with preventive practices and attitudes towards various control measures implemented in live poultry markets against H7N9.

**Results:**

Only 46.1% of the respondents recognized risks associated with contacts with bird secretions or droppings, and only 22.9% perceived personally "likely/very likely" to contract H7N9 infection. Around 60% of the respondents complied with hand-washing and wearing gloves, and only 20% reported wearing face masks. Only 16.3% of the respondents agreed on introducing central slaughtering of poultry. Being younger, involving in slaughtering poultry, having longer working hours, less access to H7N9-related information and poorer knowledge, and perceiving lower personal susceptibility to H7N9 infection were negatively associated with preventive practices. Comparing with previous studies conducted when human cases of H5N1 avian influenza infection was first identified in Guangdong, LPTs' perceived susceptibility to novel influenza viruses increased significantly but acceptance for central slaughtering of poultry remained low.

**Conclusions:**

Information on avian influenza provided through multiple communication tools may be necessary to promote knowledge among poultry traders. Familiarity with risk may have led to the lower perceived vulnerability to avian influenza and less protective actions among the LPTs particularly for those involving more risky exposure to live poultry. Reasons for the consistently low acceptance for central slaughtering of poultry await further exploration.

**Electronic supplementary material:**

The online version of this article (doi:10.1186/s12879-014-0554-8) contains supplementary material, which is available to authorized users.

## Background

A novel avian influenza A(H7N9) caused an outbreak in Mainland China in April and May, 2013 [1],[2] and resurged in winter, 2014 [3]. In Guangdong this novel influenza virus was first isolated from chicken samples in Dongguan City in April 2013, and again in May 2013 in a live poultry market (LPM) in Guangzhou, the capital city of Guangdong. The first laboratory-confirmed human case of influenza A(H7N9) infection in Guangdong reported in August 2013, was a LPM worker involved in slaughtering poultry. By the end of 2013, a total of five human cases had been reported in Guangdong [4] and by the end of February 2014, the number of confirmed H7N9 human cases in Mainland China doubled the numbers reported in the first wave outbreak between March and May, 2013 [3].

More than 70% of laboratory-confirmed human cases reported recent exposure to poultry before disease onset and 5% of them had occupational exposure to poultry [1]. There was little evidence for human-to-human transmissibility of the virus [5], but LPMs were suspected to be a major source of human infection [1],[6]. Closure of LPMs in several affected cities was shown to effectively reduce the number of new cases [6].

Live poultry traders (LPTs) have regular contacts with poultry during stocking, selling, slaughtering and de-feathering of poultry, and cleaning associated waste products. This places LPTs at increased probability of exposure to H7N9 and other poultry-related viruses compared to the general public. Therefore, LPTs are recommended to adhere to good hygiene practices, such as wearing protective clothing and eye protection, wearing face masks and receiving seasonal influenza vaccination annually [7]. However, LPTs tend to lack accurate knowledge of avian influenza and adopt inadequate protective measures when handling poultry [8]-[10], further increasing their risk of infection. There have been several studies conducted among LPTs to investigate their knowledge, attitudes and protective practices in relation to H5N1 [8]-[15]. However, the situation is unclear for H7N9 which is low pathogenic to poultry but can cause serious illness in human. Also, repeated isolation of this novel subtype avian H7N9 influenza virus from the LPMs could lead to heightened awareness of the risk associated with contacts with live poultry among the LPTs. Hence this cross-sectional study was conducted shortly after H7N9 virus was first isolated from chicken sold in one LPM of Guangzhou to investigate the knowledge, attitudes and practices of LPTs in Guangzhou towards preventive measures and potential control measures in LPMs against influenza A(H7N9). We also compared the results of current study with two earlier studies in relation to H5N1 conducted in two other cities of Guangdong Province [12],[13].

## Methods

### Study population and sample collection

Guangzhou is located in southeast China with a population of 12.8 million served by 672 registered retail LPMs across its 12 city districts. LPTs who worked in the LPMs were selected through multi-stage cluster sampling between mid-May to June, 2013, shortly after the first isolation of influenza A(H7N9) virus in poultry in Guangzhou. Initially, two LPMs were randomly selected from each district in Guangzhou. Then from each selected LPM, all workers with occupation requiring transportation of live poultry from wholesale markets to the retail outlets, selling live poultry or slaughtering of poultry for the customers were invited for the interview. Other inclusion criteria were having worked in the selected LPMs for at least three months prior to the survey, ability to understand Putonghua or Cantonese, aged above 15 years old and willing to participate in the survey. If sampling two LPMs within a particular district obtained less than 20 respondents, one additional LPM within that district was sampled to increase the pool of potential respondents for interview.

Trained interviewers from the Guangzhou Center for Disease Control and Prevention (CDC) and officials from district CDCs, with the help of LPM administrators, approached identified eligible LPT’s who met the inclusion criteria to introduce the purposes of the study, potential benefits and costs involved to eligible participants. Eligible participants were also reassured that all data collected in the study would be anonymous. Thereafter, trained interviewers approached the potential participants for the interview based on a paper-based questionnaire. All interviews were conducted during the working hours (8:30 am-17:00 pm) when the LPTs were available. Peak hours of poultry sales were avoided to improve the quality of the interviews.

The questionnaire was constructed based on similar surveys conducted in Guangzhou and other cities of China about risk perception of influenza A/H5N1 [11]-[13],[16] in the local language to assess their knowledge, attitudes and practices relating to H7N9. The questionnaire covered six main areas including knowledge (six questions) and sources of information (one question) on H7N9, perceived risk of H7N9 and attitudes towards H7N9-related preventive measures (13 questions) and the reasons for choosing negative answers (five questions), protective behaviors related to poultry exposure (eight questions), reasons for not taking preventive measure (one question), and finally demographic information. The specific questions and response scales for major study measures were detailed below.

#### Knowledge of H7N9 avian influenza and sources of information

Respondents were asked about their knowledge on the current H7N9 epidemic situation in Guangdong, correct way of handling sick or dead poultry, the transmissibility of H7N9, early symptoms of H7N9 infection in human, risk factors for H7N9 infection and vaccine availability for preventing H7N9 infection (six items). Responses for these knowledge items were "yes" or "no". A score of "1" was administrated for correctly answering each question. An additional question was included about the sources of their knowledge on H7N9.

#### Perceived risk of H7N9 and attitudes towards H7N9-related preventive measures

Respondents were ask about the severity of H7N9 relative to SARS, their likelihood of contracting H7N9 infection due to their occupational exposure, perceived confidence in preventing oneself from H7N9 infection, attitudes towards government's control measures and dissemination of H7N9-related information, attitudes towards implementation of potential control measures in wet markets including market rest days, ban on overnight storage of live poultry in retail outlets and completely closure of LPMs, and their acceptability to a vaccine for preventing H7N9 infection if it is available. Responses were generally indicated on five point Likert-type ordinal scales.

#### Protective behaviors related to poultry exposure

Respondents were asked about their frequency (always/frequently/sometimes/never) of adopting the following protective measures when working in the LPMs: wearing gloves, wearing aprons/aprons/outer garments/coveralls, wearing boots/boot covers, wearing hair covers, wearing a face mask, washing hands with soap and water after touching poultry and disinfecting hands with alcohol-based sanitizers after touching poultry. They were also asked whether they had received seasonal influenza vaccine or not over one year prior to the survey.

### Ethics

The study protocol was reviewed and approved by the Guangzhou Center for Disease Control and Prevention, China, including its ethical aspect considering the target population with low literacy. We provided an information sheet of the study and verbally described its content to potential participants. We also described the public health importance of the study and ensured anonymity of the interview. Written consent was not sought for due to expected low literacy (less than 25% attended secondary schools or above) among LPTs, but verbal informed consent was obtained from all participants before the interview.

### Statistical analysis

Descriptive analyses were initially performed to calculate proportions for each categorical variable to assess knowledge, attitudes and practices of the respondents towards H7N9. Hierarchical logistic regression models were then performed to examine factors associated with (1) adoption of preventive practices against H7N9 and (2) attitudes towards implementation of control measures in the LPMs against avian influenza. Separate hierarchical regression models were fitted to explain the above two outcomes by entering blocks of variables sequentially: Step 1, demographics; Step 2, knowledge and information access; and Step 3, attitudinal variables. To assess the level of adoption of different preventive practices, we count the number of responses with "always", "frequently" or "sometimes" for each respondent. Based on seven preventive practices, a score of 6 a cut-off point to distinguish "good preventive practices" (score ≥6) and "poor/insufficient preventive practices" (score <6). All variables were entered using the forced entry method and significances of the variable blocks were assessed by likelihood ratio tests. Adjusted odds ratios and the corresponding 95% confidence intervals for the variables were computed. Attitude and preventive behavior against avian influenza from earlier studies were compared using two-sample proportion test. All statistical tests were two-sided, with p-values of less than 0.05 considered to be statistically significant. All analyses were conducted using SPSS 17.0 [17].

## Results

**Figure 1:**
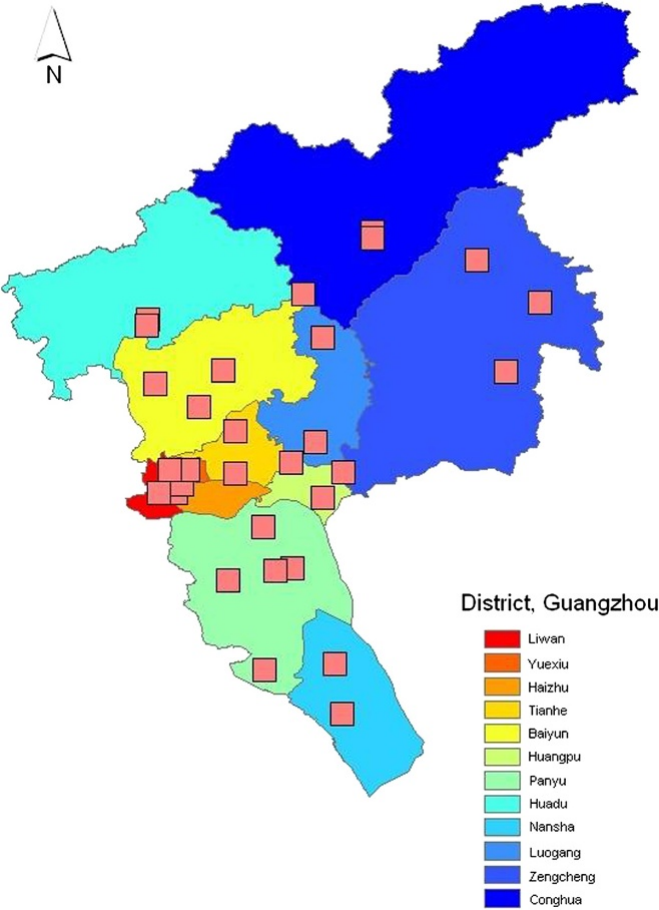
**Geographical distribution of the 38 participating live poultry markets from the 12 administrative regions in Guangzhou.**

A total of 306 poultry workers from 38 retail LPMs were contacted and interviewed from May to June, 2013 (Figure 1). A response rate of 100% was achieved with the help of LPM administrators. A median number of seven LPTs (range: 3-17) were recruited from each selected LPM. The characteristics of the respondents were shown in Table 1. Most of the respondents had low education attainment (primary or below) and had monthly income less than US$493. Almost all respondents had contact with poultry for eight hours or longer each working day and most were occupationally required to slaughter live poultry.

**Table 1:** **Socio-demographics of the respondents (N = 306)**

Variables	N	%^a^
**Demographics**		
Gender (Female)	167	54.6
Age group		
15-34	87	28.4
35-54	198	64.7
≥55	21	6.9
Education		
Illiterate	76	24.8
Primary	155	50.7
Secondary or above	75	24.5
Monthly personal income (RMB)		
<1000	35	11.4
1000-3000	174	56.9
>3000	97	31.7
Years of work in poultry industry		
<4	76	24.8
4-10	154	50.3
>10	76	24.8
Daily average hours of contact with live poultry		
<8	11	3.6
8-10	163	53.3
>10	132	43.1
Residential location		
Urban	182	59.5
Rural	124	40.5
Involved poultry slaughtering (Yes)	267	87.3
**Information sources and knowledge of H7N9**		
Sources of information		
Television	263	86.0
Newspaper	155	50.7
Internet	64	20.9
Leaflets/booklets	59	19.3
Radio	51	16.7
Friends/relatives	50	16.3
Doctor	26	8.5
Number of information sources		
1	117	38.2
2-3	145	41.4
4 or above	44	14.4
Knowledge items (proportions answering correctly)		
There is no reported H7N9 human cases in Guangdong Province (before June 30, 2013)	240	78.4
Sick or dead birds should be buried or burned	238	77.8
H7N9 is not transmissible from human to human^b^	237	77.5
Fever, cough and sore throat are early symptoms of H7N9 infection	218	71.2
Contacts with bird secretions or droppings high risk for H7N9 infection	141	46.1
Human H7N9 vaccine is not yet available	134	43.8
Answer correctly in		
All 6 items	23	7.5
5 items	91	29.7
3-4 items	157	51.3
0-2 items	55	11.4
**Perception and attitude**		
Perceived severity of H7N9 compared with SARS		
Much less/less	178	58.2
Almost the same	58	19.0
More/much more	70	22.9
Perceived personal susceptibility to H7N9 infection		
Very unlikely/unlikely	191	62.4
Neutral	45	14.7
Likely/very likely	70	22.9
Agree/strongly agree in:		
Confident in protecting myself against avian influenza infection	229	74.8
The government has good control over the epidemic	244	79.7
Being satisfied with the current control measures	230	75.2
Information is disseminated transparently and timely	267	87.3
Willing to receive H7N9 vaccine if available		
No	129	42.2
Unsure	48	15.7
Yes	129	42.2
Willing to receive free H7N9 vaccine if available (n = 177)^c^		
No	88	49.7
Unsure	33	18.6
Yes	56	31.6
Agree/strongly agree with:		
Central slaughtering of poultry	50	16.3
Regular market rest days in LPMs	179	58.5
Cleaning and disinfecting the premises, cages and other instruments of the stalls during market rest days (for applicable respondents, n = 222)^d^	214	96.4
Removing or slaughtering all birds in the stall during market rest days for cleaning and disinfecting (for applicable respondents, n = 219)^e^	124	56.6
Overnight storage of live poultry less than 10% of ]the daily wholesale volume	84	27.5

^a^May not add up to 100% due to rounding.

^b^Epidemiological evidence at the time of the survey suggested H7N9 is not transmissible between humans.

^c^The question was only asked for respondents who reported "unsure" or "not willing" to accept H7N9 vaccine if one is available.

^d^The question was only asked for respondents who reported "somewhat agree" or "agree/strongly agree" to the implementation of regular market rest days in LPM.

^e^The question was only asked for respondents who reported "somewhat agree" or "agree/strongly agree" to the implementation of cleaning and disinfecting the premises, cages and other instruments of the stalls during market rest days.

Most respondents obtained information on H7N9 from traditional media such as television or newspaper, with only 20.9% citing the internet and more than one third using only a single source (Table 1). More than 20% of the respondents incorrectly thought that Guangdong had reported H7N9 human cases at the time of the survey. More than 70% of the respondents knew that early symptoms of H7N9 influenza infection include cough, fever and sore throat, but more that 50% were unaware of the risks associated with contact with bird secretions or droppings, and the absence of a H7N9 vaccine. 29.7% correctly answered five or more items. There were no statistically significant sex or age differences (chi-square test, p-values =0.959 and 0.509 respectively) on the proportions who answered five or more items correctly concerning knowledge on H7N9.

Around 60% of the respondents perceived themselves to have low susceptibility to H7N9 infection and perceived H7N9 to have a lower severity compared to SARS (Table 1). Over 70% expressed confidence in protecting themselves from infection, and also in governmental control over the epidemic. Over 80% indicated satisfaction with the dissemination of H7N9-related information by the government.

42.2% of the respondents expressed willingness to receive human H7N9 vaccine if available, and among those who were unwilling or unsure to take the H7N9 vaccine, an addition of 31.6% are willing to take the vaccine if it was provided free of charge. Only 16.3% of the respondents supported central slaughtering for poultry and 27.5% supported limiting overnight storage of poultry. However, 58.5% of the respondents supported regular market rest days in LPMs. Of these, 56.6% supported removal or slaughtering all unsold poultry during the rest days. The major reported reasons for opposing central slaughtering of poultry included belief that "buying live poultry is a traditional habit" and "lower meat quality of chilled or frozen poultry" while "concern about the impacts on business" was the major reason for opposing implementation of regular market rest days and limiting overnight storage of poultry.

**Figure 2:**
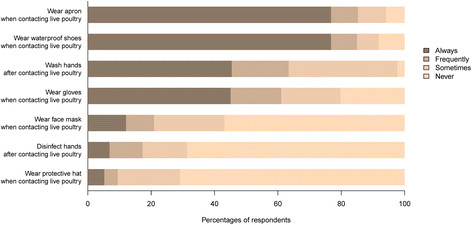
**Compliance of protective behaviors related to poultry exposure among the respondents.**

There was high compliance (over 80% for "usually" or "always") to wearing aprons and waterproof shoes when handling live poultry, but compliance to washing hands and wearing gloves decreased to about 60%, and to only 20% for wearing face masks (Figure 2). Only 9.8% of the respondents reportedly received seasonal influenza vaccine in the year preceding the survey.

The total score of the seven preventive practices (Figure 2) ranged from 0-7 with a median score of 4. Table 2 shows factors associated with LPTs’ preventive practices against H7N9 based on the hierarchical logistic regression model.

**Table 2:** **Factors associated with preventive practices against H7N9. Hierarchical logistic regression was used to include block of variables sequentially**

Independent variables			Association with preventive practices against H7N9 (aOR (95% CI))		
	N	%	Step 1 (social-demographics)	Step 2 (knowledge)	Step 3 (attitudes)
Sex					
Male	139	45.4	ref	ref	ref
Female	167	54.6	0.96 (0.53,1.72)	1.27 (0.63, 2.57)	1.66 (0.76,3.62)
Age (y)					
15-24	87	28.4	0.76 (0.38,1.54)	0.72 (0.30, 1.71)	0.72 (0.27,1.90)
35-54	198	64.7	ref	ref	ref
≥55	21	6.9	**8.12 (2.63, 25.07)** ^***^	**14.19 (3.61, 55.77)** ^**^	**13.18 (2.75,63.06)** ^**^
Education					
Illiterate	76	24.8	**0.26 (0.11, 0.57)** ^**^	**0.32 (0.11, 0.92)** ^*^	0.35 (0.11,1.08)
Primary	155	50.7	ref	ref	ref
Secondary or above	75	24.5	0.72 (0.36,1.45)	0.53 (0.23,1.22)	0.61 (0.25, 1.49)
Income (CY)					
<1000	35	11.4	ref	ref	ref
1000-3000	174	56.9	1.04 (0.41,2.69)	1.24 (0.40,3.81)	1.25 (0.35,4.40)
>3000	97	31.7	1.28 (0.47,3.50)	1.98 (0.59,6.69)	1.47 (0.38,5.66)
Area					
Rural	124	40.5	ref	ref	ref
Urban	182	59.5	**0.38 (0.21,0.69)** ^**^	0.60 (0.29,1.22)	0.72 (0.31, 1.64)
Years of working activity					
<4	76	24.8	ref	ref	ref
4-10	154	50.3	1.22 (0.58,2.55)	0.77 (0.32,1.87)	0.80 (0.31, 2.05)
>10	76	24.8	0.57 (0.23,1.44)	0.53 (0.18,1.53)	0.76 (0.23, 2.49)
Daily average hours of contact with living poultry					
<8	11	3.6	ref	ref	ref
8-10	163	53.3	0.60 (0.16,2.32)	1.11 (0.22,5.49)	0.63 (0.13,3.20)
>10	132	43.1	**0.13 (0.03, 0.55)** ^*^	0.28 (0.05,1.42)	**0.15 (0.03,0.80)** ^*^
Involved poultry slaughtering					
No	39	12.8	ref	ref	ref
Yes	267	87.5	**0.16 (0.06, 0.38)** ^***^	**0.19 (0.06,0.65)** ^**^	**0.22 (0.05,0.88)** ^*^
Number of sources to access information on H7N9					
1	117	38.2	**-**	ref	ref
2-3	145	47.4	-	**2.41 (1.06, 5.45)** ^*^	2.42 (0.95,6.17)
4 or above	44	14.4	**-**	**24.0 (5.95, 96.52)** ^***^	**25.92 (5.18, 129.66)** ^***^
With accurate knowledge in					
There is no reported H7N9 human cases in Guangdong Province (before June 30, 2013)	240	78.4	**-**	1.65 (0.67,4.04)	2.16 (0.78, 5.98)
Sick or dead birds should be buried or burned	238	77.8	**-**	**8.96 (2.59,31.08)** ^**^	**9.55 (2.41, 37.82)** ^**^
H7N9 is not transmissible from human to human	237	77.5	**-**	**3.31 (1.28, 8.57)** ^*^	**3.66 (1.23, 10.87)** ^*^
Fever, cough and sore throat are early symptoms of H7N9 infection	218	71.2	**-**	0.42 (0.16,1.09)	0.41 (0.14,1.20)
Contacts with bird secretions or feces is a high risk for H7N9 infection	141	46.1	-	**2.37 (1.12, 5.03)** ^*^	2.20 (0.96, 5.03)
Human H7N9 vaccine is not yet available	134	43.8	**-**	0.92 (0.45,1.91)	1.20 (0.53,2.70)
Perceived severity of H7N9 compared with SARS					
Much less/less	178	58.2	**-**	**-**	ref
Almost the same	58	19.0	**-**	**-**	1.55 (0.48, 5.03)
More/much more	70	22.9	**-**	**-**	0.74 (0.28, 1.97)
Perceived personal susceptibility to H7N9 infection					
Very unlikely/unlikely	191	62.4	**-**	**-**	ref
Neutral	45	14.7	**-**	**-**	**6.41 (2.26, 18.19)** ^***^
Likely/very likely	70	22.9	**-**	**-**	**4.73 (1.76, 12.70)** ^**^
Agree/strongly agree^a^ in:					
Confident in self-protection	229	74.8	**-**	**-**	1.46 (0.55, 3.90)
Transparent and timely dissemination of information	267	87.3	**-**	**-**	0.74 (0.23,2.43)
Good control over the epidemic by the government	244	79.7	**-**	**-**	0.53 (0.17,1.65)
Satisfied with the current control measures	230	75.2	**-**	**-**	1.88 (0.56,6.36)
**Model summary**					
G(-2Log likelihood)			−300.6	-224.9	-200.0
p-value^b^				<0.001	0.004
Cox & Snell R Square			0.249	0.414	0.459
ΔR^2^				0.165	0.045

aOR: adjusted odds ratio; CY: Chinese Yuan (1 CY = 0.1469); US dollar; CI: confidence interval.

^*^p < 0.05, ^**^p < 0.01 ^***^p < 0.001.

^a^Compared to "strongly disagree", "disagree" and "somewhat agree".

^b^Log-likelihood test for the significance of the factor, comparing to the previous step.

In Step 1, being aged 55 years or above was significantly associated with adoption of good preventive practices, while being illiterate, living in urban area, having contact with poultry for more than 11 hours per day and occupationally involved in slaughtering poultry were associated with poor/insufficient preventive practices. In Step 2, after controlling for socio-demographics, the number of information sources on H7N9, correctly knowing that "sick or dead birds should be buried or burned", "H7N9 is not transmissible from human to human" and "contacts with bird secretions or droppings is a high risk for H7N9 infection" were positively associated with good preventive practices. In Step 3, after controlling for demographics and knowledge factors, only perceived high personal susceptibility to H7N9 was significantly associated with good preventive practices.

Similar logistic regression models were fitted to examine factors associated with LPTs" attitudes towards implementation of control measures against avian influenza outbreaks in LPMs. These included "central slaughtering of poultry", "regular market rest days" and "overnight storage of live poultry less than 10% of the daily wholesale volume". Responses indicating agreement ("agree" or "strongly agree") in any of the three items were coded as "1", otherwise coded as "0". Table 3 showed that all three factors influenced LPTs' attitudes towards market interventions. Being occupationally required to slaughter poultry was significantly associated with lower support for the potential control measures in Step 1 while number of sources used to access information on H7N9 in Step 2 and perceived personal susceptibility to H7N9 infection in Step 3 were significantly and positively associated with support for the potential control measures.

**Table 3:** **Factors associated with attitudes towards implementation of control measures for reducing risk of avian influenza outbreaks in live poultry markets**

Independent variables			Association with attitudes towards control measures (aOR (95%CI))		
	N	%	Step 1 (social-demographics)	Step 2 (knowledge)	Step 3 (attitudes)
Sex					
Male	139	45.4	ref	ref	ref
Female	167	54.6	0.60 (0.36, 1.01)	0.66 (0.38, 1.14)	0.65 (0.37, 1.15)
Age (y)					
15-24	87	28.4	0.75 (0.40, 1.40)	0.73 (0.38, 1.42)	0.99 (0.48, 2.05)
35-54	198	64.7	ref	ref	ref
≥55	21	6.9	1.29 (0.47, 3.60)	1.40 (0.49, 3.99)	1.03 (0.33, 3.21)
Education					
Illiterate	76	24.8	0.69 (0.37, 1.27)	0.90 (0.40, 1.77)	0.95 (0.47, 1.94)
Primary	155	50.7	ref	ref	ref
Secondary or above	75	24.5	0.93 (0.49, 1.77)	0.86 (0.43, 1.70)	0.96 (0.46, 2.00)
Income (CY)					
<1000	35	11.4	ref	ref	ref
1000-3000	174	56.9	0.87 (0.40, 1.88)	0.86 (0.38, 1.93)	0.99 (0.41, 2.35)
>3000	97	31.7	1.80 (0.77, 4.23)	1.94 (0.79, 4.74)	1.92 (0.74, 4. 98)
Area					
Urban	182	59.5	ref	ref	ref
Rural	124	40.5	0.89 (0.53, 1.51)	1.02 (0.58, 1.77)	0.99 (0.54, 1.82)
Years of working activity					
< 4	76	24.8	ref	ref	ref
4-10	154	50.3	0.93 (0.48, 1.80)	0.86 (0.43, 1.74)	0.99 (0.47, 2.09)
>10	76	24.8	0.52 (0.24, 1.12)	0.60 (0.27, 1.35)	1.08 (0.45, 2.60)
Daily average hours of contact with living poultry					
<8	11	3.6	ref	ref	ref
8-10	163	53.3	1.63 (0.43, 6.19)	0.86 (0.43, 1.74)	1.29 (0.29, 5.70)
>10	132	43.1	1.40 (0.37, 5.34)	0.60 (0.27, 1.35)	1.50 (0.33, 6.73)
Involved poultry slaughtering					
No	39	12.8	ref	ref	ref
Yes	267	87.5	**0.22 (0.08, 0.62)** ^**^	**0.33 (0.11, 0.96)** ^*^	0.45 (0.15, 1.39)
Number of sources to access information on H7N9					
1	117	38.2	**-**	ref	ref
2-3	145	47.4	**-**	1.08 (0.60, 1.94)	0.94 (0.50, 1.78)
4 or above	44	14.4	**-**	**6.42 (1.65, 24.91)** ^**^	**5.06 (1.21, 21.11)** ^*^
With accurate knowledge in					
There is no reported H7N9 human cases in Guangdong Province (before June 30, 2013)	240	78.4	**-**	0.83 (0.40, 1.71)	0.95 (0.45, 2.01)
Sick or dead birds should be buried or burned	238	77.8	**-**	1.70 (0.87, 3.32)	1.56 (0.76, 3.18)
H7N9 is not transmissible from human to human	237	77.5	**-**	1.22 (0.61, 2.44)	1.19 (0.57, 2.47)
Fever, cough and sore throat are early symptoms of H7N9 infection	218	71.2	**-**	1.52 (0.82, 2.83)	1.73 (0.87, 3.43)
Contacts with bird secretions or feces is a high risk for H7N9 infection	141	46.1	**-**	1.28 (0.71, 2.32)	1.13 (0.59, 2.15)
Human H7N9 vaccine is not yet available	134	43.8	**-**	1.36 (0.77, 2.39)	1.65 (0.90, 3.03)
Perceived severity of H7N9 compared with SARS					
Much less/less	178	58.2	**-**	**-**	ref
Almost the same	58	19.0	**-**	**-**	2.17 (0.94, 4.99)
More/much more	70	22.9	**-**	**-**	1.62 (0.82, 3.21)
Perceived personal susceptibility to H7N9 infection					
Very unlikely/unlikely	191	62.4	**-**	**-**	ref
Neutral	45	14.7	**-**	**-**	**2.95 (1.14, 7.67)** ^*^
Likely/very likely	70	22.9	**-**	**-**	**2.54 (1.03, 6.26)** ^*^
Agree/strongly agree^a^ in:					
Confident in self-protection	229	74.8	**-**	**-**	0.50 (0.22, 1.14)
Transparent and timely dissemination of information	267	87.3	**-**	**-**	0.54 (0.21, 1.44)
Good control over the epidemic by the government	244	79.7	**-**	**-**	0.92 (0.39, 2.18)
Satisfied with the current control measures	230	75.2	**-**	**-**	1.85 (0.88, 3.89)
**Model summary**					
G(−2Log likelihood)			-367.1	-343.5	-321.8
p-value^b^				0.005	0.006
Cox & Snell R Square			0.090	0.158	0.216
ΔR^2^				0.068	0.058

Hierarchical logistic regression was used to include block of variables sequentially.

aOR: adjusted odds ratio; CY: Chinese Yuan (1 CY = 0.1469); US dollar; CI: confidence interval.

^*^p < 0.05, ^**^p < 0.01, ^***^p < 0.001.

^a^Compared to "strongly disagree", "disagree" and "somewhat agree".

^b^log-likelihood test for the significance of the factor, comparing to the previous step.

We also compared the perceptions, attitudes and preventive behaviors against H7N9 to those reported against high pathogenic avian influenza viruses after the first human case of H5N1 in Guangdong province was identified in 2006, in two nearby urban cities [12],[13] (Table 4). The Comparison shows that while a significantly higher proportion of LPTs (22.9%) perceived themselves susceptible to avian influenza viruses in the current H7N9 epidemic, the acceptability of central slaughtering of poultry, or market rest days did not increase markedly. However, significantly more LPTs reported wearing apron and gloves while handling live poultry, but slightly fewer reported washing their hands or wearing face masks.

**Table 4:** **Difference in attitude and preventive behaviors against avian influenza among LPTs in the current influenza A(H7N9) epidemic compared with previous studies**[12],[13]

	Positive response (%)	
Items	This study	Previous studies[[12],[13]]
Perception and attitude		
Perceived personal susceptibility to avian influenza infection^a^	22.9	**4.1** ^**^
Central slaughtering of poultry^b^	16.3	20.1
Removing or slaughtering all birds in the stall during market rest days for cleaning and disinfecting^b^	40.5	34.0
Preventive behaviors^c^		
Washing hands after contacting live poultry^b^	63.4	**81.9** ^**^
Wearing apron when contacting live poultry^a^	85.3	**28.9** ^**^
Wearing gloves when contacting live poultry^a^	61.1	**32.0** ^**^
Wearing face mask when contacting live poultry^b^	20.9	**29.1** ^*^

^*^p < 0.05, ^**^p < 0.001, test of difference in proportions.

^a^Data of previous study was from a study of awareness and prevention of avian influenza among persons of 275 closely contacting with poultries in 2007 in Shenzhen city, Guangdong province [12].

^b^Data of previous study was from a study of knowledge, attitude and behavior about human avian influenza of 259 employees in poultry in 2006 in Dongguan city, Guangdong province [13].

^c^Among those answered "always" or "frequently".

## Discussion

Occupational exposure to live poultry is believed to be an important risk factor for avian influenza A(H5N1) and A(H7N9) infection [1]. This may be the first study to report knowledge, attitudes and practices of LPTs towards H7N9 since its emergence in China in March 2013 [18], and to compare them with earlier studies on H5N1 influenza [12],[13].

Respondents mainly relied on traditional media, such as TV and newspaper, to obtain information on H7N9, consistent with previous studies on poultry workers' sources of H5N1-related information in Mainland China [12]. While the number of information sources on H7N9 accessed was found to be positively associated with adoption of preventive practices and support for potential control measures against avian influenza, more than one third of our respondents reported obtaining information on H7N9 from a single source. The generally low education attainment among the respondents may limit their ability to obtain information on H7N9 from other sources such as the internet, leaflets or booklets, in contrast to a younger population with relatively higher education in Guangzhou [19]. Previous study in Hong Kong LPTs suggested that greater utilization of the internet and other non-traditional sources to obtain information of avian influenza was associated with better knowledge of avian influenza, while training is likely to be less effective [14]. Utilization of multiple graphical or verbal communication tools, such as on-site posters or broadcasting, may be more effective in disseminating H7N9-related information to LPTs. Moreover, television exposure is generally passive, whereas internet exposure requires more active searching for information, and this would suggest more motivation, driven by perhaps greater concerns to gather information than is the case for broadcast media sources. Internet users are also more likely to be younger.

Most respondents correctly answered the knowledge items related to H7N9 but fewer than half of them recognized the risks inherent in contact with bird secretions or droppings. This is an important education target as not only LPTs unlikely to be alerted by, and report sick or dead birds possibly indicative of A(H7N9) or other infections, but their own risk assessments must necessarily be inadequate. Given that susceptibility is an important driver of protective behaviours, LPTs are at greater risk as a result. Lower knowledge of avian influenza is reported among respondents having close contact with poultry compared with the general public [11]. Moreover, being more familiar with poultry diseases may lead to lower perceived risk of infection from poultry among the poultry workers [20]. Better knowledge was associated with higher compliance to preventive practices, consistent with previous studies [10],[15].

A direct effect of knowledge on preventive practices [21],[22] would imply benefit in highlighting the potential risk in contact with bird secretions or droppings. However, consistent evidence from a wide range of studies suggests that information alone does not reliably change risky behavior [23],[24]. Most respondents (>90%) had occupational contacts with live poultry for more than eight hours daily, and most (80%) were occupationally required to slaughter poultry for the customers, confirming that LPTs have greater theoretical exposure to H7N9 infection compared with the general public [1]. However, perceived personal susceptibility to H7N9 infection among these LPTs was generally low, indicating a low awareness or minimization of their occupational risk. Most respondents perceived H7N9 to be of lesser severity relative to SARS, despite an estimated fatality rate of 35% relative to 10% for SARS [25]. The generally high perceived confidence in self-protection against H7N9 may be related to confidence in government control over the epidemic and satisfaction with government's information dissemination [22]. Since the SARS outbreak in 2003, the Chinese government has significantly improved information dissemination and control measures for communicable disease outbreaks [26],[27]. Another possible reason for this high confidence may also probably reflect perceptual bias associated with risk (poultry) familiarity [20],[28].

Unsurprisingly, there was little support among traders for the most effective control measures involving central slaughtering of poultry, limiting overnight storage in retail outlets and regular market rest days, similar to previous studies in 2008 when influenza A(H5N1) viruses were identified in poultry [12]. Although respondents were more aware of their occupational risk associated with avian influenza infection compared with that shown in previous studies [12],[13], their support for extreme control measures such as central slaughtering of poultry remained low, possibly due to "risk fatigue" after repeated isolations of novel avian influenza subtypes from LPMs. The most effective measures are likely to impact on LPTs incomes. Understandably, traders do not want to see their economic security threatened with many having poor education and few alternative employment prospects. However, while emotive arguments about "tradition" and "taste" are common, increasing population densities and development makes such arguments unsustainable. As cold-chains become better established, providing chilled chicken in markets, as happens currently for pork and beef without complaint, should become the norm. Economic concern is likely to be the single most important barrier for implementing control measures in LPMs. When stringent measures are indicated in case of elevated viral activity among poultry or human infections, an effective compensation system should be established and effectively communicated to improve compliance among LPTs. In the medium term consideration of "buying out" traders should be made to begin to reduce the prevalence of LPM. Initial opposition will likely fade if compensation is provided.

Wearing specific protective clothing (aprons or waterproof shoes) among LPTs was generally widespread, but may be more related to protecting clothing, because compliance to more-specific infection-control hand-washing and wearing gloves was, at best, only moderate. Similarly, few LPT's interviewed reported wearing face mask when contacting with poultry. Previous studies indicate low awareness among the public of exposure risk to virus-contaminated dust from droppings or feathers raised by agitated birds during purchase [29], which may account for the low compliance to wearing face mask. However, the higher perceived risk from H7N9 infection may explain the general improvement in the compliance of personal preventive measures. Uptake of seasonal influenza vaccination was low among the LPTs. Co-infection with human and avian influenza viruses can increase risks of viral reassortment in LPTs and thus of novel influenza outbreaks.

Consistent with many other studies, younger or less-educated LPTs perceived less risk and fewer reportedly adopted preventive practices. It is worth noting that the same applied to LPTs who have longer or closer contacts with poultry. Longer years of occupational exposure to poultry was also found to be associated with H7N9 seroconversion during May to December, 2013 in a nearby city in Guangdong [30]. Key barriers for adopting protective measures among these subpopulations should be studied to improve their compliance to various preventive practices.

Compared with the studies conducted when H5N1 was first identified in Guangdong [12],[13], LPTs' perceived susceptibility to novel avian influenza viruses seemed increasing with the repeated isolation of novel subtypes of influenza from poultry. However, their acceptance for policy to control poultry sold in the LPMs remained low, possibly due to concerns about employment or economic loss which await further exploration in future studies. Compliance to some self-protective behaviors such as wearing protecting clothes increased possibly as a response to their heightened perceived vulnerability.

This is a cross-sectional survey and thereby causality cannot be inferred from the study. The sample representativeness is uncertain due to lack of data on the LPT population, but the two-stage sampling should have helped minimize selection bias. The high response rate of the study is a strength. A key concern is that the study was introduced to the LPTs with the help of LPM administrators. This may have lead to over-reporting of compliance or support to local government's LPM control policy to avoid any admonition or penalties associated with non-compliance with required hygiene practices. To minimize potential bias the respondents were reassured that all data were anonymous, and results reflected low to intermediate support in some of the implemented interventions. We were not able to contact and interview some of the LPTs during our visits to the LPMs, however they represents a more mobile group who are likely to have relatively lower exposure to poultry. Different avian influenza strains and "risk fatigue" from repeated wet market-related outbreaks are also factors that may have contributed to the differences in attitude or preventive behaviors between the current and previous studies. Lastly, differences in attitudes and preventive measures against avian influenza between this and previous studies may have been attributed to various social-contextual factors which were not further examined.

## Conclusions

In summary, LPTs have higher exposure to live poultry and avian influenza compared to the general public, and most failed to recognize contacts with bird secretions or droppings as a risk of H7N9 infection. Severity and personal susceptibility of H7N9 infection were generally underestimated among LPTs. While compliance to wearing some protective clothing was generally high, compliance to hand-washing and wearing gloves was only moderate and few reported wearing face masks. This is consistent with an explanation that familiarity with risk leads to minimization of the perceptions of that risk and reduced concerns, coupled with increased belief in the controllability of the risk and low vulnerability as a result. Under these circumstances, the chances of infection for LPTs and, by extension, the public is increased. For immediate reduction of risk, consideration should be given to a rolling policy of buy-outs of live poultry licenses leading to a reduction in the number of outlets and a gradual switch to sales of chilled as opposed to live poultry as the only viable long-term solution to these regular outbreaks.

## Authors’ original submitted files for images

Below are the links to the authors’ original submitted files for images. Authors’ original file for figure 1 Authors’ original file for figure 2
