# Supplementary figures and images for: Knowledge, attitudes and practices relating to influenza A(H7N9) risk among live poultry traders in Guangzhou City, China
Source: BMC Infect Dis. 2014 Oct 18;14:554. doi: 10.1186/s12879-014-0554-8 (PMC4210513; doi:10.1186/s12879-014-0554-8)

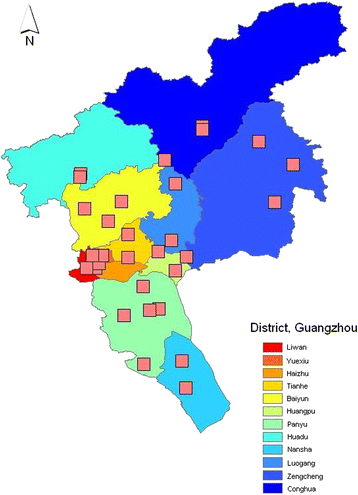

Supplement: Supplementary file 1 — Authors’ original file for figure 1 [file 12879_2014_554_MOESM1_ESM.gif]

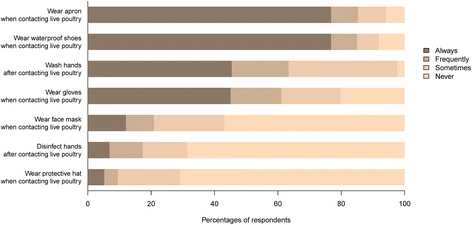

Supplement: Supplementary file 2 — Authors’ original file for figure 2 [file 12879_2014_554_MOESM2_ESM.gif]
